# Cognitive functions following initiation of antipsychotic medication in adolescents and adults at clinical high risk for psychosis: a naturalistic sub group analysis using the MATRICS consensus cognitive battery

**DOI:** 10.1186/s13034-024-00743-x

**Published:** 2024-05-04

**Authors:** TianHong Zhang, YanYan Wei, XiaoChen Tang, HuiRu Cui, LiHua Xu, YeGang Hu, YingYing Tang, Qiang Hu, HaiChun Liu, ZiXuan Wang, Tao Chen, ChunBo Li, JiJun Wang

**Affiliations:** 1 Shanghai Key Laboratory of Psychotic Disorders, Shanghai Mental Health Center, Shanghai Engineering Research Center of Intelligent Psychological Evaluation and Intervention, Shanghai Jiaotong University School of Medicine, 600 Wanping Nan Road, 200030 Shanghai, China; 2 Department of Psychiatry, ZhenJiang Mental Health Center, Zhenjiang, People’s Republic of China; 3 Department of Automation, Shanghai Jiao Tong University, 200240 Shanghai, China; 4 Shanghai Xinlianxin Psychological Counseling Center, Shanghai, China; 5 Big Data Research Lab, University of Waterloo, Waterloo, ON Canada; 6 Labor and Worklife Program, Harvard University, Cambridge, MA USA; 7 Center for Excellence in Brain Science and Intelligence Technology (CEBSIT), Chinese Academy of Science, Shanghai, People’s Republic of China; 8 Institute of Psychology and Behavioral Science, Shanghai Jiao Tong University, Shanghai, People’s Republic of China

**Keywords:** Cognition, Ultra high risk, Conversion, Schizophrenia, Prediction, Adolescent

## Abstract

**Background:**

The effects of antipsychotic (AP) medications on cognitive functions in individuals at clinical high-risk (CHR) of psychosis are poorly understood. This study compared the effects of AP treatment on cognitive improvement in CHR adolescents and adults.

**Methods:**

A total of 327 CHR participants, with an age range of 13 to 45 years, who underwent baseline neuropsychological assessments and a 1-year clinical follow-up were included. Participants with CHR were categorized into four groups based on their age: adolescents (aged < 18) and adults (aged ≥ 18), as well as their antipsychotic medication status (AP+ or AP−). Therefore, the four groups were defined as Adolescent-AP−, Adolescent-AP+, Adult-AP−, and Adult-AP+.

**Results:**

During the follow-up, 231 CHR patients received AP treatment, 94 converted to psychosis, and 161 completed the 1-year follow-up. The Adolescent-AP+ group had more positive symptoms, lower general functions, and cognitive impairments than the Adolescent-AP− group at baseline, but no significant differences were observed among adults. The Adolescent-AP+ group showed a significant increase in the risk of conversion to psychosis (*p* < 0.001) compared to the Adolescent-AP− group. The Adult-AP+ group showed a decreasing trend in the risk of conversion (*p* = 0.088) compared to the Adult-AP− group. The Adolescent-AP− group had greater improvement in general functions (*p* < 0.001), neuropsychological assessment battery mazes (*p* = 0.025), and brief visuospatial memory test-revised (*p* = 0.020), as well as a greater decrease in positive symptoms (*p* < 0.001) at follow-up compared to the Adolescent-AP+ group. No significant differences were observed among adults.

**Conclusions:**

Early use of AP was not associated with a positive effect on cognitive function in CHR adolescents. Instead, the absence of AP treatment was associated with better cognitive recovery, suggesting that AP exposure might not be the preferred choice for cognitive recovery in CHR adolescents, but may be more reasonable for use in adults.

## Introduction

Cognitive impairment is common in patients with psychosis and has been well documented in previous studies [1, 2]. These impairments not only exist in the chronic stages of psychosis [3] but also have varying degrees in the early and even prodromal stages [4–6]. Cognitive impairment plays a crucial role in the development of psychotic symptoms and is strongly associated with poor functional outcomes. Unfortunately, cognitive impairment remains an unmet therapeutic challenge, and minimizing it during treatment is a key focus for clinicians.

Since antipsychotic drugs (AP) remain the first-line treatment for patients with psychosis, their effects on cognitive function have attracted widespread attention. However, the results of these studies are often inconsistent. Some studies [7, 8] have suggested that AP, especially second-generation AP, might have beneficial effects on cognitive function in patients with chronic psychosis [9]. Contrasting findings from real-world follow-up studies [10, 11] have indicated that psychosis exposure to AP has a negative effect on verbal learning and memory performance over time, suggesting that prolonged or higher-dose antipsychotic use is associated with adverse cognitive outcomes. In addition, naturalistic follow-up studies [12, 13] on the discontinuation of AP showed that discontinuation might be beneficial for cognitive function. However, Singh et al. [14] reported that reducing the dose of AP during the maintenance phase was associated with improved cognitive function without an increased risk of relapse, implying that dose reduction may be better than discontinuation.

Most studies on the cognitive impact of APs have been conducted in adult patients in whom the illness has progressed to the maintenance or chronic stages. These findings make it difficult to determine whether the effects of AP on cognitive function follow a similar pattern in the prodromal phase of psychosis, that is, clinical high risk (CHR), in which individuals are often adolescents. Our previous study [15] found that compared with adult CHR, cognitive functions in adolescents at CHR showed more significant impairments and were associated with a higher risk of conversion to psychosis. How initiating AP treatment at the CHR stage of psychosis affects the trajectory of cognitive function in adolescents remains largely unknown. Given the large number of adolescents with CHR who receive AP treatment in real-world clinical practice in our sample [16–18], it is important to establish whether there is a neutral, beneficial, or harmful association between AP treatment and cognitive functioning.

We used an ongoing longitudinal program that included a 12-year naturalistic CHR cohort to assess and compare the effects of APs on cognitive function between adolescents and adults in the CHR phase of psychosis. Specifically, our aims were as follows: (1) to compare the cognitive performance between adolescents and adults who were treated with or without AP during the 1-year follow-up; (2) to compare the cognitive changes between adolescents with CHR and adults treated with or without AP; and (3) to examine the differences in the effects of AP on cognitive functions and clinical outcomes between adolescents and adults.

## Methods

### Study design and setting

Current data were collected from an ongoing longitudinal study of the ShangHai At Risk for Psychosis-extended (SHARP-extended) program [19–21] conducted between 2016 and 2021. CHR participants enrolled in a clinical risk assessment and intervention for early psychosis program that was implemented at the Shanghai Mental Health Center (SMHC) in China, which is China’s largest outpatient medication-management and psychotherapy-providing mental health clinic. The participants in this study did not receive psychotropic medications. They did not have any history of substance abuse or dependence according to the specific exclusion criteria. The research ethics committee of SMHC approved this study (IRB2016-009). All participants provided written informed consent during study recruitment. Participants < 18 years of age had their consent forms signed by their parents and youths.

Three research assistants conducted the follow-ups. Individuals with CHR were followed up every 3 months through phone conversations on their medical condition and medication intake. Participants were told that they could contact the research assistants at any time to answer the questions. At the 1-year follow-up, participants were invited back for face-to-face interviews and cognitive function reassessment. The clinical outcome determination was based mainly on 1-year face-to-face interviews (out of 327 CHR individuals, 207 had at least one face-to-face interview during the follow-up), partly on telephone interviews of CHR individuals or their caregivers, and on the medical information confirmed using clinician reports and medical records.

### Sample

This study included a subset of the main study (*n* = 400) consisting of participants who had completed at least baseline neuropsychological assessments and a 1-year clinical follow-up (*n* = 327). The participants had an age range of 13 to 45 years, with adolescents defined as those aged < 18 and adults as those aged ≥ 18. The mean ± standard deviation (SD) age was 18.8 ± 5.0 years and 177 (54.1%) were female. During follow-up, 231 CHR were treated with AP, and 94 (28.7%) converted to psychosis. Among them, 161 (49.2%) completed the 1-year follow-up neuropsychological reassessment. Inclusion criteria were: age 13–45 years; fulfilling the diagnostic criteria for one of three psychosis risk syndromes: (1) attenuated positive symptom syndrome; (2) brief intermittent psychotic syndrome or (3) genetic risk and deterioration syndrome as defined using the Structural Interview for Prodromal Syndromes (SIPS) interview [22]; no current or lifetime psychotic episode; the symptoms are not better explained by other non-psychotic disorders or substance abuse disorder; no a past usage of psychotropic medication, regardless of dosage; no present or past history of psychoactive drugs (e.g. methamphetamine, etc.); no known neurological or endocrine disorders; no mental retardation; sufficient mastery of mandarin; and have the ability to understand and sign an informed consent form.

### Measurements

Face-to-face interviews were conducted using the SIPS [22] to identify individuals with CHR syndrome. In our previous studies [23, 24], the Chinese version of the SIPS [25], which was developed by our team demonstrated good inter-rater reliability (intraclass correlation coefficient: *r* = 0.96, *p* < 0.01; SIPS total score) and validity (26.4% of the subjects converted to psychosis in the following 2 years) in China. The first author received SIPS certification at a Yale University-sponsored SIPS training course and has developed extensive expertise in its use by managing clinical assessments since the initiation of the original SHARP project [26–28]. Structured clinical interviews were conducted with three senior psychiatrists who had completed the training required for this type of investigation. The inter-rater reliability for the SIPS ranged from 0.80 to 0.92 among the ratings of the trained interviewers.

Neurocognitive assessments were applied using the Chinese version of the Measurement and Treatment Research to Improve Cognition in Schizophrenia (MATRICS) consensus cognitive battery (MCCB) [29–31]. The MCCB was administered according to standardized guidelines provided in the test manual. The Chinese version of the following eight subtests were included in the present study: (1) Part A of the Trail Making Test (Trail Making A), (2) Symbol Coding of the Brief Assessment of Cognition in Schizophrenia (BACS) (BACS symbol coding), (3) Category Fluency Test (Category Fluency), (4) Continuous Performance Test-Identical Pairs (CPT-IP), (5) Spatial Span of the Wechsler Memory Scale-III (WMS-3 spatial span), (6) Revised Hopkins Verbal Learning Test (HVLT-R), (7) Revised Brief Visuospatial Memory Test (BVMT-R), and (8) Neuropsychological Assessment Battery: Mazes (NAB mazes). Test-retest reliability in a previous Chinese psychosis sample ranged from 0.73 to 0.94 [31]. Notably, except for the Trail Making A test, higher scores on the other tests indicated better performance.

The MCCB was selected as the primary cognitive assessment tool due to its widespread use in clinical research settings and comprehensive evaluation of cognitive function across multiple domains relevant to schizophrenia. MCCB assessments were conducted in a quiet and independent room, with operators and participants engaging face-to-face according to the MCCB operator’s manual. Operators underwent training in administering the MCCB cognitive tests and successfully completed assessments to ensure consistent administration. The entire testing session typically lasted approximately 1 h.

### Medication exposure

The use of AP was examined every 2 months via telephone and 1-year face-to-face follow-ups by asking about the participants’ medication history. Depending on whether AP was administered for at least 2 weeks during the follow-up period, the sample was divided into AP and AP+ groups. Using an olanzapine-equivalent dose of AP [32], the mean dosage in the AP+ group was 8.5 (SD = 6.1) mg/day, and the mean duration of administration was 36.2 (SD = 20.0) weeks. Among the 231 CHR participants who were treated with antipsychotics, there were 101 individuals treated with Aripiprazole (43.7%), 89 with Olanzapine (38.5%), 30 with Amisulpride (13.0%), and 11 with Risperidone (4.8%). A small group of participants took psychoactive medication other than AP (67 individuals took antidepressants, with a fluoxetine-equivalent [33] dose of 22.9 [SD = 13.0] mg/day, and the mean duration for which it was taken was 34.0 [SD = 19.5] weeks). Additionally, 59 CHR individuals were treated with benzodiazepines, and 37 had taken traditional Chinese medicine at some point during the study period.

### Data analysis

SPSS for Windows (version 20.0; IBM, Armonk, NY, USA) was used for data analysis. Statistical significance was set at *p* < 0.05. Participants with CHR were divided into four groups (Adolescent-AP−, Adolescent-AP+, Adult-AP−, and Adult-AP+) based on their age (Adolescent, 13–17 years), age (Adult, 18–45 years), and whether AP was taken. Quantitative variables are expressed as mean (SD) and qualitative variables as frequencies (%). Independent *t* tests were conducted to measure AP− vs. AP+ group differences in continuous variables, and Chi-square statistics were used to examine categorical variables. Baseline and follow-up means and change from baseline to follow-up within each group (Adolescent-AP−, Adolescent-AP+, Adult-AP−, and Adult-AP+ groups) were analyzed separately using a paired sample *t* test. The Kaplan–Meier method and log-rank tests were used to plot survival curves and for comparisons between the AP and AP+ groups, further stratified by adolescents and adults. Repeated measures analysis of variance (RMANOVA) was performed on the AP+, AP−, Adolescent and Adult groups to estimate and compare the trajectories of clinical features and neurocognitive performances. RMANOVA with a factorial design (2 time points × 2 statuses) was performed to determine significant interactions between the groups (AP− vs. AP+, Adolescent vs. Adult) in terms of their effects on clinical features and neurocognitive performance.

## Results

### Sample baseline characteristics

The participants, aged between 13 and 45 years, were categorized as adolescents (< 18 years old) and adults (≥ 18 years old). Within the adolescent group, the individuals in the Adolescent-AP+ and Adolescent-AP− groups did not differ in terms of demographic variables. The Adolescent-AP+ group had significantly higher positive symptom scores and lower baseline global assessment of function (GAF) scores than the Adolescent-AP− group. Individuals in the Adolescent-AP+ group performed significantly worse than those in the Adolescent-AP− group on HVLT-R, NAB mazes, Category Fluency and CPT-IP at baseline. Within the adult group, individuals in the Adult-AP+ and Adult-AP− groups did not differ in terms of demographic and baseline clinical variables or cognitive performances (Table 1).

**Table 1:** Baseline demographic, clinical and cognitive variables in adolescents and adults at clinical high risk for psychosis, comparison between the Adolescent-AP+ vs. Adolescent-AP− groups, and Adult-AP+ vs. Adult-AP− groups

Variables	Adolescent-AP−	Adolescent -AP+	AP− VS. AP+		Adult-AP−	Adult-AP+	AP− VS. AP+	
			t/χ^2^	*p*			t/χ^2^	*p*
Cases (*n*)	61	153	–	–	35	78	–	–
Age (years)	15.9 (1.6)	15.9 (1.4)	*t* = 0.001	0.999	24.8 (4.6)	24.2 (4.4)	*t* = 0.688	0.493
Male [n (%)]	26 (42.6)	73 (47.7)	*χ*^*2*^ = 0.454	0.500	17 (48.6)	34 (43.6)	*χ*^*2*^ = 0.242	0.623
Education (years) [mean (S.D.)]	9.3 (1.8)	9.3 (1.4)	*t* = 0.033	0.974	13.0 (2.2)	13.0 (3.2)	*t* = 0.016	0.987
Father education [mean (S.D.)]	11.1 (3.5)	10.8 (3.9)	*t* = 0.592	0.555	9.8 (4.1)	9.7 (3.8)	*t* = 0.118	0.906
Mother education [mean (S.D.)]	10.4 (4.3)	10.1 (3.9)	*t* = 0.424	0.672	9.3 (3.8)	9.7 (4.0)	*t* = 0.465	0.643
Family history (none) [n (%)]	54 (88.5)	14 (9.2)	*χ*^*2*^ = 2.587	0.274	3 (8.6)	4 (5.1)	*χ*^*2*^ = 1.321	0.517
Family history (low-risk) [n (%)]	5 (8.2)	16 (10.5)			2 (5.7)	9 (11.5)		
Family history (high-risk) [n (%)]	2 (3.3)	123 (80.4)			30 (85.7)	65 (88.3)		
Before GAF	78.0 (3.3)	78.7 (4.5)	*t* = 1.092	0.276	79.0 (3.0)	77.6 (5.0)	*t* = 1.595	0.114
Baseline GAF	56.2 (7.6)	53.8 (7.3)	*t* = 2.180	**0.030**	54.5 (7.4)	55.4 (7.5)	*t* = 0.590	0.556
Positive symptoms	9.2 (2.9)	10.7 (3.6)	*t* = 2.828	**0.005**	9.3 (3.4)	9.4 (3.3)	*t* = 0.248	0.805
Negative symptoms	11.4 (6.0)	12.8 (5.5)	*t* = 1.637	0.103	12.7 (5.7)	11.5 (6.3)	*t* = 1.007	0.316
Disorganized symptoms	6.2 (3.1)	6.8 (3.2)	*t* = 1.287	0.207	6.6 (3.2)	6.0 (2.8)	*t* = 0.912	0.364
General symptoms	8.8 (3.3)	9.1 (3.0)	*t* = 0.710	0.479	8.2 (2.9)	9.7 (2.8)	*t* = 2.598	0.011
Trail making A	31.8 (12.8)	33.3 (14.8)	*t* = 0.710	0.479	34.8 (10.7)	35.3 (12.7)	*t* = 0.190	0.850
BACS symbol coding	59.8 (10.5)	57.3 (10.0)	*t* = 1.622	0.106	57.0 (11.3)	54.4 (10.7)	*t* = 1.190	0.237
HVLT-R	25.1 (4.9)	23.4 (5.3)	*t* = 2.150	**0.033**	23.2 (5.8)	22.5 (4.6)	*t* = 0.637	0.525
WMS-3 spatial span	15.5 (3.2)	15.8 (3.1)	*t* = 0.519	0.604	15.5 (3.3)	15.1 (2.8)	*t* = 0.565	0.574
NAB mazes	19.2 (5.5)	16.2 (6.7)	*t* = 3.098	**0.002**	15.7 (6.5)	15.0 (5.9)	*t* = 0.631	0.529
BVMT-R	28.4 (5.9)	27.0 (5.8)	*t* = 1.688	0.093	24.2 (6.9)	24.3 (7.0)	*t* = 0.058	0.954
Category fluency	20.8 (4.4)	18.6 (5.3)	*t* = 2.855	**0.005**	20.6 (5.3)	19.4 (5.7)	*t* = 1.079	0.283
CPT-IP	2.6 (0.7)	2.4 (0.8)	*t* = 2.078	**0.039**	2.7 (0.9)	2.4 (0.9)	*t* = 1.609	0.110

Bold in significant

*AP−* treated without antipsychotic medication, *AP+* treated with antipsychotic medication, *BACS* brief assessment of cognition in Schizophrenia symbol coding, *BVMT-R* brief visuospatial memory test-revised, *CPT-IP* continuous performance test-identical pairs, *HVLT-R* Hopkins verbal learning test-revised, *GAF* global assessment of function, *Before GAF* the highest GAF score in the past year from the baseline, *Baseline GAF* GAF score at baseline, *None family history* having no family members with mental disorders, *Low-risk family history* a first-degree relative with non-psychotic disorders, *High-risk family history* having at least one first-degree relative with psychosis, *NAB* neuropsychological assessment battery mazes, *WMS-3* Wechsler memory scale–third edition spatial span

### Sample follow-up characteristics

Within the adolescent group, individuals in the Adolescent-AP+ group had significantly lower follow-up GAF scores than the Adolescent-AP− group. Individuals in the Adolescent-AP+ group performed significantly worse than those in the Adolescent-AP− group on BVMT-R and Category Fluency at follow-up. Within the adult group, individuals in the Adult-AP+ and Adult-AP− groups did not differ in follow-up clinical variables and cognitive performances (Table 2).

**Table 2:** Follow-up clinical and cognitive variables in adolescents and adults at clinical high risk for psychosis who completed the 1-year reassessment of cognitive tests, comparison between AP+ and AP−

Variables	Adolescent-AP−	Adolescent-AP+	AP− VS. AP+		Adult-AP−	Adult-AP+	AP− VS. AP+	
			t	*p*			t/χ^2^	*p*
Cases [*n*]	10	104	–	–	9	38	–	–
Follow-up GAF	73.7 (6.9)	66.4 (10.0)	2.247	**0.027**	68.8 (10.2)	70.8 (7.7)	0.660	0.513
Positive symptoms	2.9 (2.1)	5.0 (4.1)	1.602	0.112	5.0 (3.8)	3.4 (3.6)	1.191	0.240
Trail making A	25.9 (6.2)	29.4 (11.9)	0.924	0.357	30.9 (13.6)	30.3 (10.5)	0.151	0.880
BACS symbol coding	60.6 (15.0)	55.4 (9.6)	1.545	0.125	57.9 (18.5)	56.2 (13.0)	0.330	0.743
HVLT-R	26.2 (4.0)	23.6 (4.9)	1.635	0.105	23.1 (4.1)	23.8 (4.5)	0.426	0.672
WMS-3 spatial span	16.9 (2.5)	16.3 (2.8)	0.657	0.512	14.7 (3.6)	16.0 (3.2)	1.094	0.280
NAB mazes	21.3 (5.7)	16.9 (7.1)	1.898	0.060	15.0 (8.9)	15.4 (5.9)	0.151	0.881
BVMT-R	31.5 (3.2)	26.9 (5.9)	2.450	**0.016**	22.8 (7.5)	25.6 (6.1)	1.201	0.236
Category fluency	22.5 (4.0)	18.4 (5.2)	2.414	**0.017**	21.6 (7.0)	19.6 (5.1)	0.952	0.346
CPT-IP	2.5 (0.8)	2.6 (0.8)	0.142	0.888	3.0 (0.7)	2.6 (0.8)	1.541	0.130

Bold in significant

*AP−* treated without antipsychotic medication, *AP+* treated with antipsychotic medication, *BACS* brief assessment of cognition in Schizophrenia symbol coding, *BVMT-R* brief visuospatial memory test-revised, *CPT-IP* continuous performance test-identical pairs, *HVLT-R* Hopkins verbal learning test-revised, *NAB* neuropsychological assessment battery mazes, *WMS-3* Wechsler memory scale–third edition spatial span

### Self-controlled comparisons of neurocognitive performances

**Fig. 1:**
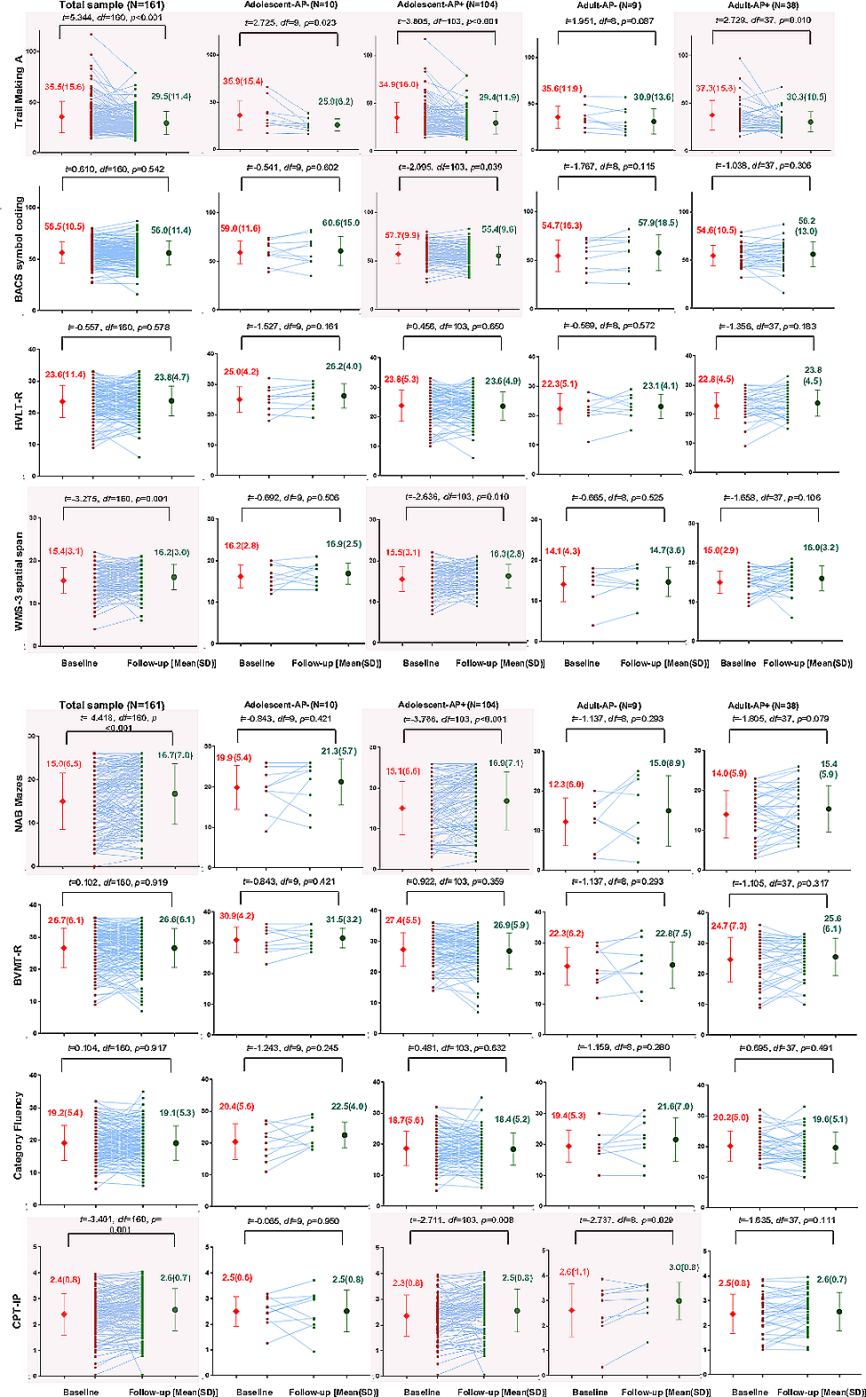
Profile and paired tests for comparisons of neurocognitive changes in Trail Making A, BACS symbol coding, HVLT-R, WMS-3 spatial span, NAB mazes, BVMT-R, Category Fluency, CPT-IP in overall, Adolescent-AP−, Adolescent-AP+, Adult-AP− and Adult-AP+ groups. *AP−* treated without antipsychotic medication, *AP+* treated with antipsychotic medication, *BACS* brief assessment of cognition in Schizophrenia symbol coding, *BVMT-R* brief visuospatial memory test-revised, *CPT-IP* continuous performance test-identical pairs, *HVLT-R* Hopkins verbal learning test-revised, *NAB* neuropsychological assessment battery mazes, *WMS-3* Wechsler memory scale–third edition spatial span. Bold in significant

Overall, the paired *t*-test sample (Fig. 1) showed that the trail making A (*p* < 0.001), WMS-3 spatial span (*p* = 0.001), NAB mazes (*p* < 0.001), and CPT-IP (*p* = 0.001) subtest performances improved significantly. In the Adolescent-AP− group, performance on the trail making A test (*p* = 0.023) improved significantly. In the Adolescent-AP+ group, the performance on the trail making A (*p* < 0.001), BACS symbol coding (*p* = 0.039), WMS-3 spatial span (*p* = 0.010), NAB mazes (*p* < 0.001), and CPT-IP (*p* = 0.008) subtest performances improved significantly. In the Adult-AP− group, performance on the CPT-IP test (*p* = 0.029) improved significantly. In the Adult-AP+ group, performance on the trail making A test (*p* = 0.010) improved significantly.

### Antipsychotic taken and conversion

**Fig. 2:**
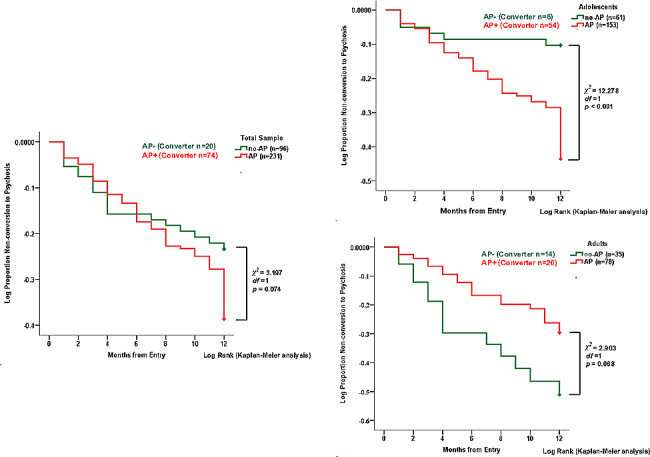
Kaplan–Meier curves for overall survival, CHR adolescents and CHR adults, by comparison (log-rank test) between AP− and AP+ groups at the end point of follow-up. *AP−* treated without antipsychotic medication, *AP+* treated with antipsychotic medication, *CHR* clinical high risk for psychosis. *AP−* treated without antipsychotic medication, *AP+* treated with antipsychotic medication, *CHR* clinical high risk for psychosis

Overall, compared with the AP− group, CHR individuals in the AP+ group showed a significant increasing trend for risk of conversion to psychosis (*p* = 0.074). In adolescent CHR, compared with the AP− group, participants in AP+ group showed a significant increase in the risk of conversion to psychosis (*p* < 0.001). In adult CHR, compared with the AP− group, participants in the AP+ group showed a significantly decreasing trend in the risk of conversion to psychosis (*p* = 0.088) (Fig. 2).

### Changes in clinical characteristics and cognitive performances

**Fig. 3:**
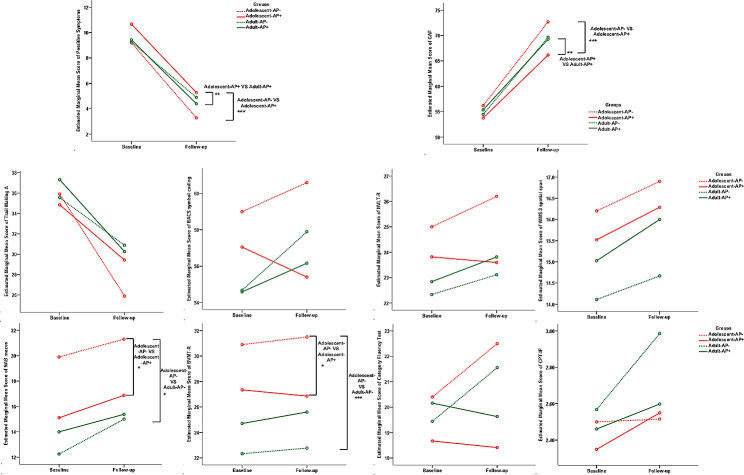
Mean score trajectories for clinical features and neurocognitive performances based on the repeated-measures analysis of variance, compared between AP− and AP+, Ado and Adu groups at baseline and follow-up. *AP−* treated without antipsychotic medication, *AP+* treated with antipsychotic medication, *BACS* brief assessment of cognition in Schizophrenia symbol coding, *BVMT-R* brief visuospatial memory test-revised, *CPT-IP* continuous performance test-identical pairs, *HVLT-R* Hopkins verbal learning test-revised, *NAB* neuropsychological assessment battery mazes, *WMS-3* Wechsler memory scale–third edition spatial span. *p < 0.05; **p < 0.01; ***p < 0.001

In adolescent CHR, the mean increase in the GAF score (*p* < 0.001), NAB mazes (*p* = 0.025) and BVMT-R (*p* = 0.020) and the mean decrease in the positive symptoms score (*p* < 0.001), were greater for the AP− group than in the AP+ group at the 1-year follow-up (Tables 3; Fig. 3). In the adult CHR group, no significant differences were evident in clinical and cognitive variables between the AP and AP+ groups. In the AP− group, the mean increase in the NAB mazes (*p* = 0.018) and BVMT-R (*p* < 0.001) were greater for the adolescent group than in the adult group. In the AP+ group, the mean increase in the GAF score (*p* = 0.007) and the mean decrease in the positive symptom score (*p* = 0.006) were greater in the adult group than in the adolescent group.

**Table 3:** Mean changes of clinical features and neurocognitive performances in AP+ and AP−, Adolescent and Adult groups at baseline and follow-up. Repeated-measures analysis of variance shows the difference in change over time between the groups

Subtests	Adolescent		Adult		AP−		AP+	
	AP− VS AP+		AP− VS AP+		Adolescent VS Adult		Adolescent VS Adult	
Variables	Estimated change difference	Between groups(*p*)	Estimated change difference	Between groups(*p*)	Estimated change difference	Between groups(*p*)	Estimated change difference	Between groups(*p*)
GAF	4.5 (0.9)	**< 0.001**	− 0.3 (1.3)	0.840	2.4 (1.3)	0.73	− 2.4 (0.9)	**0.007**
Positive symptoms	− 1.7 (0.4)	**< 0.001**	0.2 (0.6)	0.773	− 0.8 (0.6)	0.153	1.1 (0.4)	**0.006**
Trail making A	− 1.2 (3.9)	0.749	− 0.6 (4.3)	0.896	− 2.3 (5.4)	0.666	− 1.7 (2.2)	0.458
BACS symbol coding	3.6 (3.4)	0.291	0.9 (3.8)	0.659	3.5 (4.7)	0.453	0.9 (1.9)	0.659
HVLT-R	1.9 (1.4)	0.184	− 0.6 (1.6)	0.703	2.9 (2.0)	0.146	0.4 (0.8)	0.643
WMS-3 spatial span	0.6 (0.5)	0.452	− 1.1 (1.0)	0.243	2.2 (1.2)	0.071	0.4 (0.5)	0.427
NAB mazes	4.6 (2.0)	**0.025**	− 1.1 (2.4)	0.658	7.0 (2.9)	**0.018**	1.3 (1.2)	0.273
BVMT-R	4.1 (1.7)	**0.020**	− 2.6 (1.9)	0.183	8.6 (2.4)	**< 0.001**	1.9 (1.0)	0.053
Category fluency	2.9 (1.5)	0.059	0.6 (1.7)	0.724	1.0 (2.1)	0.655	− 1.4 (0.9)	0.125
CPT-IP	0.1 (0.2)	0.811	0.2 (0.3)	0.391	− 0.3 (0.4)	0.443	− 0.1 (0.1)	0.573

Bold in significant

*AP−* treated without antipsychotic medication, *AP+* treated with antipsychotic medication, *BACS* brief assessment of cognition in Schizophrenia symbol coding, *BVMT-R* brief visuospatial memory test-revised, *CPT-IP* continuous performance test-identical pairs, *HVLT-R* Hopkins verbal learning test-revised, *NAB* neuropsychological assessment battery mazes, *WMS-3* Wechsler memory scale–third edition spatial span

## Discussion

### Key findings

In this study, we prospectively investigated cognitive function in a large sample of adolescents and adults at the CHR stage in the pre-morbid phase of psychosis who did and did not receive AP over a 1-year period. To the best of our knowledge, this is the first study to compare the effects of APs on cognitive function between adolescents and adults in a CHR population. The main finding was that initiating AP treatment in adolescents with CHR increases the risk of conversion to psychosis and results in poorer symptomatic, functional, and cognitive recovery compared with initiating AP treatment in adults with CHR. In our previous study [15], cognitive functions in adolescents with CHR showed more significant impairments and were associated with a higher risk of conversion to psychosis compared to CHR adults. The current findings add new evidence that the early use of AP in adolescents with CHR may negatively affect their cognitive function recovery, leading to an increased risk of conversion.

### AP− versus AP+

We found that 71.5% of adolescents and 69.0% of adults at the CHR stage of psychosis were treated for AP during the 1-year follow-up period. The approximate 70% AP exposure rate in our sample is in line with our prior investigations [17, 21, 34] of the AP assumption in the Chinese CHR population between 2011 and 2016, which showed that 68–72% of CHR individuals initiated AP treatment at the CHR stage. However, this rate is much higher than that in other CHR studies, which reported rates between 20 and 33% [35–37]. We previously reported that AP prescriptions by clinicians and assumptions made by CHR individuals largely cohere with respect to the target positive symptoms and global functions [16]. Consistently, this study found that AP+ group had a significantly higher level of positive symptoms, poorer baseline global functions, and worse cognitive functions than those in the AP− group. Uniquely, this AP exposure pattern is only found in CHR adolescents.

Using linear mixed models, we found that those in the CHR without AP treatment group, the adolescent group improved significantly more compared with the adult group in the NAB mazes and BVMT-R tasks. For the conversion outcome, no significant difference was detected between the AP− and AP+ groups in terms of conversion rate, but a trend found to be significant was that those in the AP+ group were at an increased risk for psychosis than those in the AP− group. This result is in accordance with that of a previous meta-analysis [38] showing that baseline exposure to AP is associated with a higher risk of conversion to psychosis in comparison with antipsychotic-naïve individuals. Importantly, this study only supported this association in adolescents with CHR, although the opposite trend was found in adults with CHR, in which the Adu-AP+ group showed beneficial effects on symptomatic and functional improvements and tended to have a lower risk of conversion than those in the Adu-AP− group. This may be an important clue for clinicians to be particularly vigilant about regarding AP prescriptions in adolescents with CHR.

### Adolescents versus adults

The comparison of the effects of APs on cognitive function between adolescents and adults in the CHR groups separately reflects the age effects in the two groups. The patterns of the effects of AP on cognitive function seemed to vary in the adolescent group compared to the adult group. We found that the NAB mazes and BVMT-R tasks differed significantly between the AP and AP+ groups, and that adolescents without AP treatment performed better than those treated with AP. Our findings revealed that the effect of AP on cognitive improvement was diverse in different age groups and not balanced across all domains. A possible explanation for such diverse AP effects between adolescent and adult participants with CHR may be the differences in the trajectory of neuropsychological development [39, 40]. For example, previous studies have shown that cognitive functions, such as executive functions and other more complex tasks, do not mature until early school years, adolescence, or even early adulthood [41, 42]. Therefore, premature use of AP in adolescents may have a negative impact on neural networking, plasticity and cognitive development [43].

### Cognitive trajectory

The cognitive trajectories of the performances of the NAB maze and BVMT-R tasks were significantly different between the adolescent and adult groups in individuals with CHR without AP treatment and between the AP and AP+ groups in adolescents with CHR. Compared to other tests, these two tests may be more complex and may be included in the reasoning, problem-solving, and visuospatial learning domains of cognition, representing executive functioning and working memory abilities [44–46]. Our previous studies have suggested that executive functioning and working memory abilities are particularly valuable in capturing CHR states and predicting psychosis [6, 15, 47, 48]. Considering the central role of the impairment of visuospatial learning and working memory abilities in the development of psychosis from the CHR stage, especially for adolescents [15], the results of this study may further suggest the underlying reasons for the negative effects of AP usage in CHR adolescents.

### Limitations

This study has a few limitations. First, the sample was recruited from a single site; although it has the advantage of homogeneity, the generalizability of the findings is limited. Second, it is important to note that the SHARP-extended cohort was surveyed naturalistically, and the number of individuals with CHR who were not exposed to AP was significantly lower than that of those treated with AP. However, this reflects the prescription patterns, and not the design. Third, a small proportion of individuals with CHR were treated with antidepressants, benzodiazepines, and other psychotropic medications, which may have confounded the findings. Fourth, our AP data may have been subject to inaccuracies and potential recall biases. We performed tripartite checks involving individuals with CHR, family members, and medical records to confirm their medical details. However, these approaches are less accurate than other strict methods, such as pill counts.

Additionally, it is important to acknowledge the challenge of distinguishing between false positives and false negatives regarding the impact of AP on cognition. While high doses of AP may indeed have a negative effect on cognition, separating this from cognitive decline driven solely by the underlying disease poses a significant challenge. False positives, individuals who exhibit minimal cognitive decline despite increased AP doses, may exist because they are unlikely to progress through the disease regardless of AP adjustments. Conversely, false negatives, who experience more pronounced cognitive decline despite low AP doses, may be predisposed to develop psychosis regardless of AP usage. This inherent complexity in differentiating AP effects from psychosis progression should be considered when interpreting the findings of this study.

Furthermore, the study is based on naturalistically collected data with a non-random assignment of individuals with CHR to AP medication. It cannot be excluded, and it is actually likely, that clinicians prescribed AP to those individuals with CHR who presented with more worrisome clinical and/or functional features and who therefore were at greater risk of a worse outcome. Under these conditions, it is not possible to draw causal inferences, but only to point to suggestive associations. Thus, the conclusions regarding the effect of early AP use on cognitive function should be interpreted with caution.

Finally, it is important to note that the exploratory nature of this study and the limited sample size may have constrained our ability to further explore the differential impact of AP on cognitive function between more detailed age groups, as the continuum of physiological and psychological changes associated with aging is continuous. We may not have captured nuances in the relationship between AP use and cognitive outcomes, such as specific ages at which the effects become more pronounced.

### Future directions

We acknowledge that the 1-year follow-up period in our study may limit our ability to capture individuals who conversion to psychosis after this time frame. As such, the impact of AP on long-term cognitive outcomes remains uncertain. We recognize the importance of conducting long-term follow-up studies to address this limitation. Future research endeavors should consider extending the follow-up period to at least 2–3 years to comprehensively assess the sustained effects of AP on cognitive function. Additionally, detailed analysis of cognitive function changes in patients following the conversion to psychosis is warranted. By incorporating these considerations into study design, we can better elucidate the relationship between AP use and cognitive outcomes in individuals at risk for psychosis. Furthermore, in future studies, we also aim to conduct more rigorously controlled clinical trials to further investigate the relationship between AP dose and changes in cognitive function among individuals at CHR. Specifically, we plan to implement detailed documentation of medication doses, types, durations, and adherence levels to allow for a more nuanced analysis of medication effects. By conducting clinical trials with stricter control over AP use, we hope to elucidate whether there is a linear relationship between AP dose and cognitive function changes in this population.

Not only will we explore the linear relationship between AP dose and cognitive function in future studies, but we will also consider the possibility of a non-linear relationship between AP dose and changes in cognitive function among individuals at CHR. Drawing from past research by Andreasen’s group [49, 50], which has explored the relationship between AP dose and brain volume in schizophrenia, we recognize the importance of investigating potential adverse effects that may emerge at moderate doses of AP. Therefore, we plan to incorporate analyses that explore the possibility of non-linear effects of AP dose on cognitive outcomes in CHR individuals. By examining dose-response relationships and potential threshold effects, we can better inform clinical decision-making regarding the optimal use of AP to mitigate cognitive decline in this population. Additionally, we will explore potential moderators and mediators of these relationships to provide a more comprehensive understanding of the factors influencing AP effects on cognitive function.

## Conclusion

Initiating AP treatment in adolescents with CHR is associated with less improvement in cognitive recovery than initiating AP treatment in adults with CHR, potentially leading to an increased risk of conversion to psychosis and poorer symptomatic and functional recovery. Given that cognitive function serves as a crucial predictor of conversion and functional outcomes, clinicians must carefully weigh the decision to initiate AP treatment in adolescent CHR individuals against the potential impact on cognitive recovery. Furthermore, there may be a need to exercise even greater caution when initiating AP treatment in this population and to explore the provision of alternative therapeutic non-pharmacological strategies [51].

## Data Availability

No datasets were generated or analysed during the current study.

## Abbreviations

AP: Antipsychotic
BACS: Symbol coding of the brief assessment of cognition in Schizophrenia
BVMT-R: Revised brief visuospatial memory test
CHR: Clinical high-risk
CPT-IP: Continuous performance test-identical pairs
GAF: Global assessment of function
HVLT-R: Revised Hopkins verbal learning test
MCCB: Measurement and treatment research to improve cognition in Schizophrenia (MATRICS) consensus cognitive battery
NAB: Neuropsychological assessment battery
SD: Standard deviation
SHARP-extended: Shanghai at risk for psychosis-extended
SIPS: Structural interview for prodromal syndromes
SMHC: Shanghai mental health center
WMS-3: Wechsler memory scale-III
